# War both reduced and increased inequality over the past ten thousand years

**DOI:** 10.1073/pnas.2400695121

**Published:** 2025-04-14

**Authors:** Mark D. McCoy, Jennifer Birch, Shadreck Chirikure, Pablo Cruz, Adam S. Green, Detlef Gronenborn, Dan Lawrence, Paul Roscoe

**Affiliations:** ^a^Department of Anthropology, Florida State University, Tallahassee, FL 32304; ^b^Department of Anthropology, University of Georgia, Athens, GA 30602; ^c^Research Laboratory for Archaeology and the History of Art, School of Archaeology, University of Oxford, Oxford OX1 3TG, United Kingdom; ^d^Unidad Ejecutora en Ciencias Sociales Regionales y Humanidades, Consejo Nacional de Investigaciones Científicas y Técnicas, Buenos Aires 4600, Argentina; ^e^Department of Archaeology, University of York, York YO1 7EP, United Kingdom; ^f^Department of Environment and Geography, University of York, York YO1 7EP, United Kingdom; ^g^Leibniz-Zentrum für Archäologie, Mainz D-55116, Germany; ^h^Department of Archaeology, Durham University, Durham DH1 3LE, United Kingdom; ^i^Department of Anthropology and Climate Change Institute, University of Maine, Orono, ME 04469

**Keywords:** wealth inequality, warfare, Gini coefficient

## Abstract

Scholars are divided over the long-term effects that war has had on inequality. Some have argued that conflict grows the gap between rich and poor. Others counter that violence levels out wealth differences. The GINI Project Database is a large global sample of archaeological data on house sizes created to investigate what factors influenced economic inequality over long periods of time, including warfare. Over 39,000 individual residential units were coded as having fortifications present or absent, with about a third in fortified settlements (n = 13,372) and two-thirds in unfortified settlements (n = 25,897). We compared residential disparity (differences in residential unit sizes within a settlement) at sites around the world (n = 770) dating as far back as 10,000 y ago. We found strong support for the expectation that conflict was linked with increasing residential disparity (i.e., wealth inequality), specifically when governance was less collective and the main factor limiting agricultural production was available land. However, we also found long periods, especially in the earliest eras represented in the database, when fortified settlements had residential disparity less than or equal to unfortified settlements. These early societies tended to be more collective with available labor limiting agricultural production. We speculate that in these communities, the relative value of coalition building was higher, whereas in cases where conflict was associated with rising residential disparity, elites found a way to leverage their wealth to protect property. These contradictory models help explain why war co-occurs with increasing inequality in some cases and decreasing inequality in others.

## Significance

War can, it has been argued, increase, or decrease, inequality. We examined the long-term effects of war on economic inequality in global history by focusing on archaeological evidence from fortified settlements. We find strong evidence linking conflict and increasingly unequal house sizes. Contrastingly, war also appears to have acted as a leveling mechanism in the past, especially in earlier periods. Aspects of agriculture and governance may explain why war cooccurs with increasing inequality in some cases and decreasing inequality in others. This is the largest synthesis of its kind and provides direction for future research for years to come. We also hope to encourage an evidence-based discussion of war and inequality that ultimately will promote peace and equality.

Social scientists often see war and inequality, specifically the unequal distribution of material wealth within groups, as having coevolved (1–5). Some have gone as far as to propose that war emerged as a means of procuring resources at the expense of others (e.g., refs. 6–13), thereby serving as an agent of rising inequality. At the same time, it has been suggested that war can be a leveling mechanism against rising inequality (14–16) and that it fosters prosocial behavior among those that cooperate in waging it (17, 18). The result is a situation where it is not clear whether one model is correct and the other incorrect, in an absolute sense, or if the two opposing models are each valid in certain circumstances, or if neither is a suitable foundation for a holistic understanding of the long-term effects of war on inequality.

The dissonant literature on war and inequality provides strong, if conflicting, theoretical links between each variable. Examining those links in the distant past, however, is not straightforward. Violent conflict and wealth inequality, for example, are each inherently difficult to systematically examine over the full course of human history since physical evidence from the archaeological record can be ambiguous. Trauma might represent injury or death from accidents and/or individual-scaled violence rather than war. Artifacts might have been used for hunting animals, or to project status, rather than as weapons. The goods found with burials may better index other factors than economic wealth since how people imbue objects with value is highly variable (19). Little wonder that most theories of conflict and inequality are based on documentary sources from the archives of states, empires, and colonial powers despite good evidence that conflict and inequality underwent profound changes well before the invention of writing (for a recent review see ref. 20).

Documenting the long-term cumulative effects of war on material inequality—its capacity to grow or shrink gaps between rich and poor—is challenging but not impossible. Contemporary archaeology can contribute considerably to this, and social science more broadly, thanks to, “the accumulation of considerable new fieldwork data from around the world and the development of new methods and concepts that transform our evidence into reliable reconstructions of past social dynamics” (21). In this study, we use a large and rich comparative database, called the GINI Project Database (22), to examine two archaeological proxies for wealth inequality and war: *residential disparity* (the difference between the sizes of housing within a settlement expressed as a Gini coefficient) and fortification (presence/absence). In this global dataset, we find support for both the notion that war has ratcheted up inequality in the past, and war having acted as a leveler of inequality. We speculate that the relative value of agricultural labor, governance, and other factors explain why war affects inequality one way in some cases, and the opposite way in others.

## The GINI Project

The Global Dynamics of Inequality (GINI) project was an international research effort, inspired by previous examples in ecology (see ref. 22) that brought together disparate researchers who had data and interests relevant to the analysis of economic inequality over the long term. GINI began by selecting scholars who responded to a widely circulated call-for-interest and then grew to include some 22 collaborators (23, 24). The group as a whole designed the general guidelines for what would be appropriate existing data, a procedure for coding data, and quality review. We report here on a small part of the results of this collaborative synthesis (see other papers in the Special Feature).

Our proxy for inequality is *residential disparity*—the difference between the sizes (m^2^) of residential units (i.e., housing) within a settlement expressed as a Gini coefficient (highest possible disparity = 1.000; no disparity = 0.000). We cannot overstate the importance of regional expertise in creating this proxy. The project assigned regional experts to find data, determine whether it was fit for purpose, and oversee its coding. We outline these decisions in area-specific metadata published in the *Journal of Open Archaeology Data* (JOAD). This methodological strategy reflects a consensus among archaeologists that the best way to measure, and understand, inequality in the past is with contextual knowledge specific to a time period, place, and culture. Thus, while we sacrifice precision by centering our discussion on a single metric (residential disparity), our collaborative research design leverages years of accumulated knowledge about individual cases.

There is no proxy for inequality in the past that is globally accepted among archaeologists. Qualitative metrics of inequality are commonplace in the literature (e.g., classification of subsets of the population as elites), but there are scholars who think attempting to quantify inequality via residential disparity is doomed to failure because it is founded on the idea that “bigger is better” when it comes to housing. To this, we counter that size difference in residences were, and are, one of the most visible, enduring, and common, cross-cultural signals of inequality. People can easily judge relative differences in the sizes of houses in their community. House size does not have the capacity to be hidden, like portable material culture, or nonmaterial wealth, like rights. Housing has intrinsic value as shelter, and that value, all other things held equal, increases with more space for people and their property (see refs. 25 and 26 for more on embodied, relational, and material wealth).

**Fig. 1.**
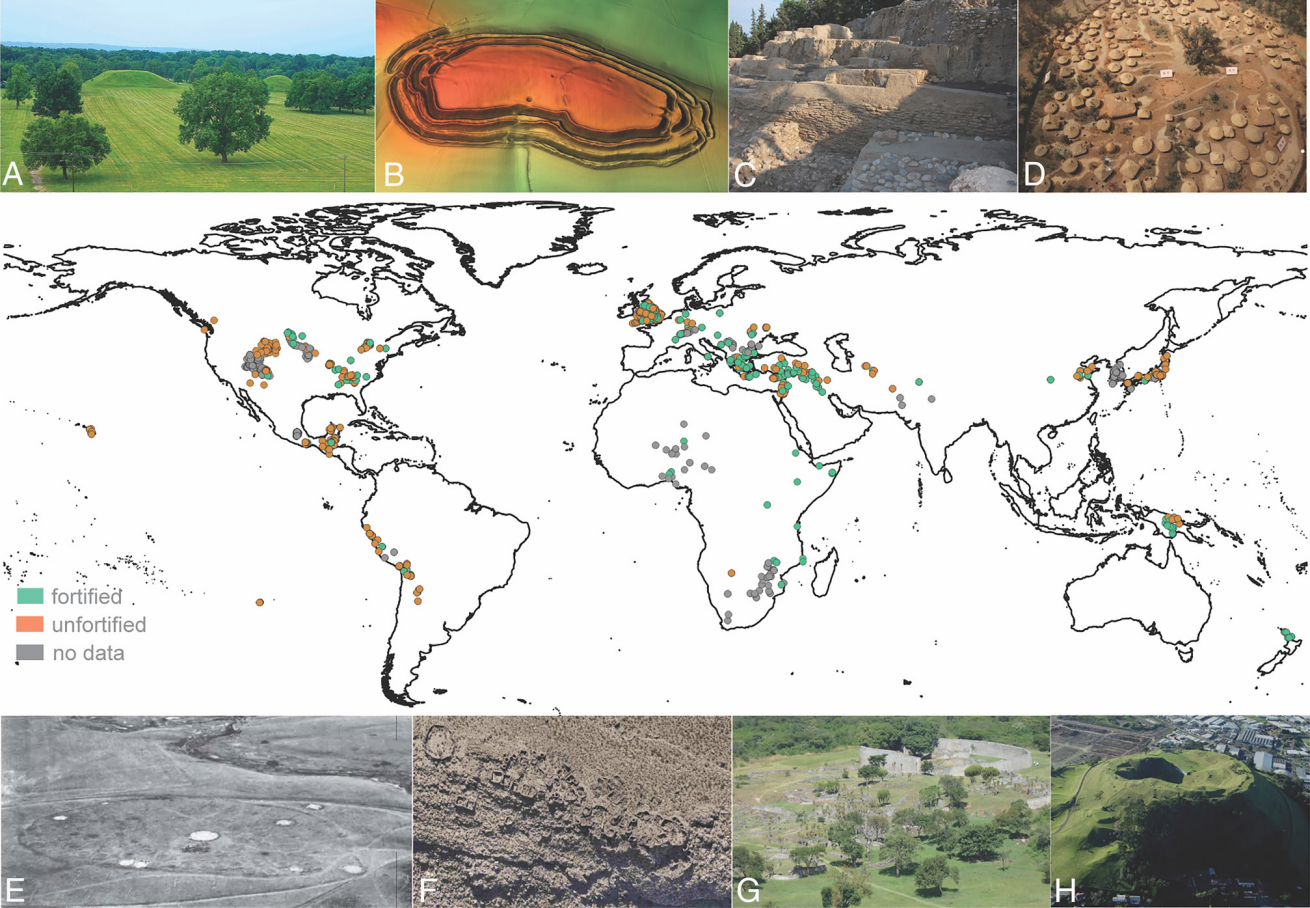
The GINI Project Database includes over 35,000 individual residential units from archaeological sites around the world including: (*A*) Cahokia (Illinois, USA), where residential disparity varied over time (Gini coefficient from 0.193 to 0.498) (Photo by Ko Hon Chiu Vincent); (*B*) remote sensing (airborne lidar) has been key to mapping many of these sites, as shown here at the Iron Age hillfort of Maiden Castle (UK) (model by Rouven Meidlinger); (*C*) Mersin-Yumuktepe, Southwest Asia (Türkiye) includes a fortified citadel surrounded by an undefended lower town (Gini coefficient: 0.456) (Photo credit: IHA), (*D*) the village of Jiangzhai (China), had a 2-m wide ditch constructed during the Banpo Phase (Gini coefficient: 0.467) (Photo of reconstructed miniature by Gary Lee Todd); (*E*) Star Village (North Dakota, USA) is a fortified Arikara village built in 1862 and is one of the most recent examples in the database (Gini coefficient: 0.279) (image source: Bureau of American Ethnology), (*F*) Jirira (Bolivia), located in the Southern Andes, is a good example of the use of natural terrain for defense (Gini coefficient: 0.213) (Photo by Pablo Cruz); (*G*) the Late Iron Age capital of Great Zimbabwe (Zimbabwe) has strong evidence for differences in wealth but little residential disparity (Gini coefficient: 0.256) (Photo by Graciela Gonzalez Brigas); (*H*) Maungarei Pa/Mt. Wellington Pa (Aotearoa/New Zealand), located in Tāmaki Makaurau/Auckland, is a hilltop fortified settlement with a residential disparity similar to others in the islands of Polynesia (Gini coefficient: 0.527) (Photo by Kevin Jones).

The GINI Project Database includes 53,466 residential units from 1,176 sites around the world (see ref. 27). The majority of these settlements had sufficient evidence to be evaluated for the presence/absence of fortifications (*SI Appendix*, Table S1). About 30% of evaluated sites, representing 13,372 residential units, were in settlements that were fortified in some fashion. They include examples from all major regions of the world and date to as far back as 10k years ago (Fig. 1).

There is no proxy for war in the past that is universally accepted among archaeologists. However, previous studies, such as Keeley et al. (28), have proposed classificatory schemes to determine categorically if a specific site was fortified. Such schemes have the advantage of being able to be unambiguously applied, but their strictness almost certainly underestimates the incidence of fortification by erasing nuances only recognizable at the local scale. There are inherent difficulties, moreover, in evaluating “defensiveness” using archaeological data alone (29). The type and scale of fortification are influenced by natural landform, the nature of warfare, available raw materials, and labor. People also value fortifications for more than purely martial reasons that can be functional (e.g., walls can prevent flooding) and/or symbolic (30, 31).

As with inequality, regional expertise was also key in devising our proxy for war. Fortifications are one of the most visible signatures of violent conflict or the threat thereof. Our identification of the presence or absence of fortifications thus reflects the consensus of archaeologists who work on that particular period, place, and culture. For example, when regional experts coded some sites as “fortified” that have no known additional human-made defenses, we accepted that classification. This flexibility, we believe, helps us avoid the misclassifications that plague any universal scheme applied to the archaeological record. While we provide all the necessary data and metadata to reconstruct and evaluate how we weighed different kinds of evidence for defenses, further research could undertake interrater reliability checks. The lack of these types of controls in archaeology has had a negative impact on reconstructing prehistory as shown in the case of false positive zooarchaeological identification of fauna exposed by subsequent ancient DNA analyses (e.g., refs. 32 and 33).

Our regional experts only coded settlements as fortified if the indicators of fortification were contemporaneous with the coresidential group whose dwellings were used to assess residential disparity. This allowed us to link our two metrics. Fortifications, in places like Aotearoa (New Zealand) and Iroquoia (North America) are among the costliest pieces of preindustrial military technology in terms of construction and maintenance (34, 35). The large size of some fortifications means that, in places like Europe, they can live on in the architecture of a city far past the period when they are a valid metric for conflict. Here again, to avoid this pitfall, we rely on regional expertise. Fortifications function to demonstrate military power. They keep enemies out and ensure that if they get in, they cannot escape with their lives, and, by maximizing both eventualities, they deter enemies from thinking of attacking in the first place (36). Thus, they are doubly important in their intrinsic value for the protection of the people and property within and potential value, as a deterrent. We acknowledge, however, that we lack fine-grained information on the nature of conflict (e.g., civil war, invasions, scale) (37) and that we do not include fortifications in nonsettlement contexts (e.g., the Great Wall of China), since we lack concurrent and clearly associated measures of residential disparity.

The GINI Project Database includes many other categorical variables, two of which, we argue, are important to contextualizing our results: 1) agricultural production as either more limited by the amount of available labor or by the amount of land for farming; 2) societies with less or more collective forms of governance. They were conceptualized and coded based on recent research (38, 39) and are explored at length in parallel studies using the project database (40, 41). We limit our analysis to two-category classifications (i.e., land or labor limited; more or less collective governance). Subsampling into three or more categories, while valuable for unpacking these variables, in our study resulted in categories with too few case studies to yield useful results.

## Expectations

Archaeology contributes a long-term perspective to social science (21). To do this effectively, Smith (42), building on Booth et al. (43), advocates that archaeologists follow an argument structure that explicitly lays out their claims or hypotheses; presents reasons to support a claim and evidence to support those reasons; acknowledges alternatives and complications; and (importantly to our study) what “warrant or principle… justifies the links among claims, reasons, and evidence.” In this study, we focus on underlying warrants. Smith identified two primary types of warrants specific to archaeology: theory and comparative data. There is currently ample theoretical justification for the notions that “war rachets up inequality,” and “war is a leveler of inequality.” While contradictory, both are logically consistent with bodies of social theory and supported by different empirical case studies. Nonetheless, they have never been examined using comparative data at the scale represented in the GINI Project Database.

The literature on war and inequality gives us two reasonable expectations when examining the GINI Project Database: either Expectation #1, less residential disparity at fortified sites than unfortified sites, or Expectation #2, more residential disparity at fortified sites than unfortified sites. We call these “expectations,” rather than hypotheses, to distinguish what we describe here (i.e., the exploration of a dataset to evaluate whether the link between war and inequality is warranted) from hypothesizing after the results are known (aka HARKing) (see ref. 42). We are not directly evaluating claims (e.g., war is a causal mechanism shaping inequality), but rather evaluating whether there is warrant in the comparative data for what already has theoretical support.

## Results

**Fig. 2.**
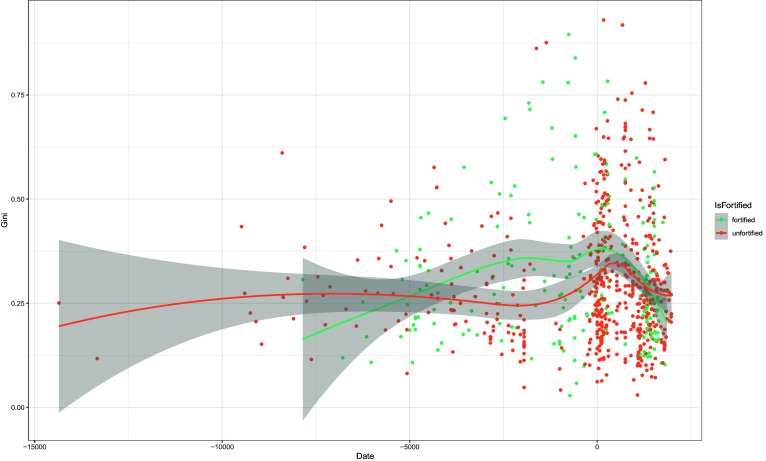
Residential disparity (Gini coefficient) in unfortified and fortified sites. Dates shown as BCE (−) and CE (+). Each point represents a settlement in a single period. 95% CI is shown (loess method).

**Fig. 3.**
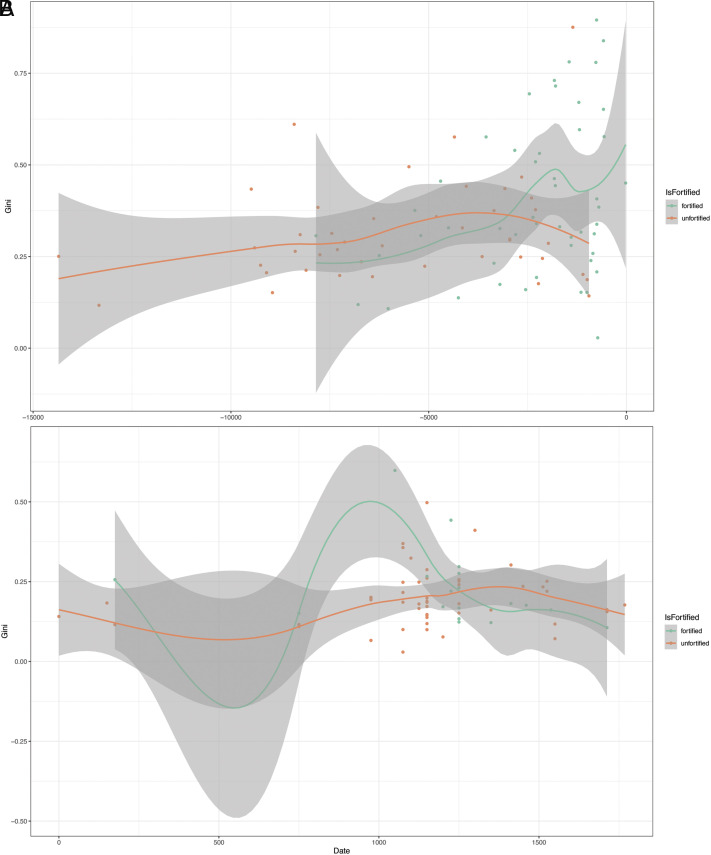
Regional case studies: (*A*) Southwest Asia and (*B*) Southeast North America. Residential disparity (Gini coefficient) is shown alongside percentage of sites with land or labor limited agricultural production, and more or less collective forms of governance. These results suggest that the onset of rising inequality with conflict may be linked to less collective governance, while its long-term longevity seems to be associated with agricultural production that is land-limited. Dates shown as BCE (−) and CE (+). Each point represents a settlement in a single period. 95% CI is shown (loess method).

We cannot reject the expectation that war had a leveling effect on residential disparity given that we find weak support for it in both hemispheres. Fig. 2 compares fortified and unfortified sites in global time series. In the earliest period when comparison is possible (10k to 6k years ago), we find fortified sites were slightly less, or equal to, unfortified sites in terms of residential disparity. It is unclear whether this weak support for Expectation #1 holds for the Western Hemisphere, or for regions of the Eastern Hemisphere where coverage is poor. In Southwest Asia (Fig. 3*A*), we begin to see fortified sites in the GINI Project Database with the onset of farming (variable: [Plant Cultivation Common]), and these fortified sites remain at equal or less residential disparity for the next 4k years. In North America, the same is true for sites from the past 700 years, long after the onset of farming, but importantly, spanning the period prior to and after Columbian contact (Fig. 3*B*).

We find strong support for the expectation of greater residential disparity at fortified sites (Expectation #2) in two regions: Southwest Asia and Southeast North America (Fig. 3). In Southwest Asia (Fig. 3*A*), the transition from Expectation #1 to #2 occurred over a 2k year period from 6250 BP (4300 BCE) to 4050 BP (2100 BCE). In Southeast North America (Fig. 3*B*), there are few datapoints prior to the upward trend in residential disparity at fortified sites; nonetheless, by around 950 BP (1000 CE), Gini coefficients rise, especially at sites like Cahokia (peak: 0.498) and Kincade (peak: 0.598) (see also ref. 40). By 700 BP (1250 CE), the pattern has reversed and fortifications remain at an equal or slightly lower level of residential disparity for the remainder of the sequence. The timing, trend, and location all point to this being part of the larger Cahokia phenomenon (*Discussion*).

### Case Studies: Southwest Asia and Southeast North America.

In the cases where we find strong support for greater residential disparity at fortified sites—Southwest Asia and Southeast North America—we examined additional variables in the GINI Project Database that might help explain why they saw a rise in inequality, and why it was long lived in one case (Southwest Asia) but short lived in the other (Southeast North America).

#### Governance and the rise of inequality.

The GINI Project Database gives us the ability to compare societies classified as “less collective” and “more collective” in terms of governance (see ref. 39 for more on this schema). We find Expectation #2 (fortified sites with higher Gini coefficient index values) is a global pattern in societies with less collective forms of governance ([PolityGov], code:0). This holds true for Southeast North America (0.200 vs. 0.270) and Southwest Asia (0.299 vs. 0.406). In the project database, we begin to consistently find examples of less collective societies in Southeast North America from 1000 CE and in Southwest Asia from around 5000 BCE, roughly at the beginning of the transition to Expectation #2. We have no reason to believe that there was just one factor linking conflict with increased residential disparity, but if there was, and it is represented in the GINI Project Database, then a less collective form of government is that factor.

Fortifications in Southwest Asia vary in layout in ways that we speculate might have implications for the transition from Expectation #1 to Expectation #2. To investigate this, we created an additional simple classification for types of fortification based on the presence/absence of defensive walls around the exterior of a settlement (single, double walls) and/or internal defensive walls around a precinct of the settlement (*SI Appendix*, Table S2). We found that in this region, the earliest examples (Expectation #1) were all settlements with single external walls. In the centuries leading up to the transition, we begin to find examples in Anatolia of double external walls at some settlements (Aşağı Pınar), internal walls at others (Mersin-Yumuktepe), and the first examples of fortifications with high levels of inequality (greater than 0.400; Hassek Höyük; Aşağı Pınar). After the transition to Expectation #2, fortified sites with internal walls have significantly higher levels of inequality (all greater than 0.550) compared with all others sites (unfortified or fortified) of the same size. We believe that these internal areas acted as citadels built to protect the people and property of the wealthiest groups from both invaders and coresidents.

#### Agriculture and the persistence of inequality.

We could point to many differences between Southwest Asia and Southeast North America, but, in our view, the one that best explains why the transition to Expectation #2 was long-lived in one case and not the other was labor-limited vs. land-limited farming. In the long-lived example of Southwest Asia, the transition begins with the first appearance in the database of societies coded as practicing land-limited farming, a condition that then becomes dominant (Fig. 3*A*). In Southeast North America, all societies are coded as labor-limited (Fig. 3*B*). Here again, we have no reason to believe that a single factor made conflict and increased residential disparity a more-or-less stable condition. If there was, however, and it is represented in the GINI Project Database, then land-limited farming is that factor.

All things being equal, if conflict mapped on to locations with higher agricultural productivity, we would expect to see it linked to larger house sizes (41, 42). We found that larger mean residential unit size (mean of variable: [TotalAreaHouse]) is indeed correlated with higher frequency of fortification in both cases that fit Expectation #2 (*SI Appendix*, Table S3). In Southwest Asia, there is an increase from sites in the smallest quartile (Q1:10% fortified) to the largest (Q4:81% fortified). We also see this in Southeast North America where we have no sites in the smallest quartile that were fortified (Q1:0% fortified) but it is common at sites with larger average residential unit size (Q4:48% fortified). There are, of course, many other factors that could account for this trend, for example, larger house size could simply be a type of defensive strategy that is covarying with the presence of fortifications.

## Discussion

*Perhaps there never was a monument more characteristic of an age and people than the Alhambra; a rugged fortress without, a voluptuous palace within; war frowning from its battlements; poetry breathing throughout the fairy architecture of its halls. One is irresistibly transported in imagination to those times when Moslem Spain was a region of light amid Christian, yet benighted Europe; externally a warrior power fighting for existence; internally a realm devoted to literature, science, and the arts; where philosophy was cultivated with passion, though wrought up into subtleties and refinements; and where the luxuries of sense were transcended by those of thought and imagination.*
  -Washington Irving, *Tales of the Alhambra* (1832)

When the author Washington Irving visited the ruins of Alhambra (a fortified settlement from the 14th century), it inspired him to see more than people protecting their lives and property. In this study, we have revisited nearly 12k residences of people who, in different locations around the world over the past 10k years, lived in a range of different kinds of fortified settlements. While our dataset has its limitations, we believe it can nonetheless speak to fundamental questions about human history.

To start, one might reasonably ask why are we looking at how war affected inequality and not the other way around? Could there be some more complex dynamic between the two or other factors, such the development of bronze metallurgy and horse-mounted warfare (26), driving both? The fact is, based on the data on hand, we cannot detangle the causal mechanism(s) at play. But we can determine whether these two variables are historically linked and cooccur in a fashion that is consistent, or inconsistent, with the diametrically opposed ways that war is hypothesized to affect inequality.

Could war have had a leveling effect on inequality? We could not find a time or place where fortified settlements were places with significantly less residential disparity than unfortified settlements. But there are many examples where fortified sites have equal or slightly less residential disparity. Therefore, we stop short of claiming to have demonstrated that conflict is not a leveler of inequality. In fact, we find that our metric for wealth inequality (Gini coefficient values based on residential unit sizes) is consistently low at fortified sites for thousands of years in the Eastern Hemisphere.

Our working explanation is that this situation was in some way tied to the premium placed on creating, maintaining, and protecting coalitions of people. All fortifications protect people, but for the societies where we see fortified sites with slightly lower Gini coefficient values—in which collective governance and labor-limited food production were commonplace—a coalition was key to survival even when people did not feel the need to build fortifications. It is not difficult to imagine that displays of residential disparity were suppressed when there was an expectation for common defense of the community. To borrow a phrase from the economist Jared Bernstein (44), people used their homes to signal the idea that “we’re in this together.”

We acknowledge that there are leveling mechanisms outside of the context of conflict (see ref. 16). For example, hunting-gathering, it has been argued, likely encouraged egalitarianism (e.g., ref. 45), although this idea has been challenged (e.g., ref. 46). Other proposed levelers—revolutions, collapses, plagues—are plausible, either as alternative or in tandem with war. The significance of our results is we have demonstrated that the idea that war was leveling inequality is warranted in comparative data, thus opening up a pathway for future hypothesis testing.

Could war have had a ratcheting-up effect on inequality? We examined two locations—Southwest Asia and Southeast North America—where the GINI Project Database supports the idea that war could have encouraged higher levels of residential disparity, with governance and agriculture as key factors. Societies coded as less collective tend to have larger residential disparity at fortified settlements, and this pattern is clearly emergent from a subset that is both less collective and land-limited in terms of agricultural production. In addition, if larger average residential unit size corresponds to higher levels of productivity (47, 48), then we have warrant to believe that conflict was often over especially productive locations. However, this pattern has other reasonable, but untested, explanations.

We speculate that what changed was how people used their wealth to protect their property (i.e., land, houses, objects). While fortifications have the potential to protect both people and their property, they would have been especially attractive to those with more wealth to protect. At some point, richer households may have worked out how to use their wealth to underwrite and improve fortifications and offset the apparent loss of coalition building provided through a collective display of equity. This would account for the strong association between fortified sites with high residential disparity and “less collective” forms of governance. For this to have persisted for as long as it did, it may have required more than just economic change; it may have demanded a fundamental ideological shift that valued property much or more than (some) human lives (for an evolutionary perspective see ref. 49). This would seem to be counter to the idea of fortifications as public goods. Fortifications that surround entire settlements certainly have that quality, but in some cases, we see more effort was made to protect a subset of residents.

New research on the evolution of “citadels” helps explain why we see this shift in ancient societies. Green et al. (50) define citadels as small fortified settlements produced by early low-growth economies. Within citadels, a relatively small subset of people extracted wealth from a substantial offsite population, perhaps by defending large accumulations of stored raw materials or produce. Citadels represent the confluence of “[f]ortifications, concentrated wealth, and monumental architecture with probable elite associations” and are closely associated with the emergence of capital in the archaeological record (50). They contrast with the world’s first cities which instead were extensive settlements with many residences, high-economic growth, and trivial levels of inequality (50). Cities with walls largely protect residences, not storage areas, and enclose entire settlements.

Both cities and citadels emerge in the millennia following the widespread adoption of agro-pastoral economies (in high-growth and low-growth economies respectfully) and do not share an evolutionary relationship. However, many cities do eventually become “citadelized,” perhaps as their economic growth slows down and/or when they are co-opted by a citadel elite. We lack the kinds of data necessary to fully test this model here, but at the beginning of the transition to higher levels of residential disparity, we do see inner defensive walls being built in Southwest Asia. In other words, citadels appear at exactly the time and place we might expect.

We have suggested that, in the case of Southeast North America, the effect of war on increasing inequality was short-lived in the absence of land-limited societies. Undoubtedly, multiple factors were at play. The establishment of Cahokia around 1000 CE introduced a period of regional peace (51), followed around 1150 CE by what might have been the Western Hemisphere’s largest investment in palisades. Cahokia’s defenses do not appear to have been breached. But there is evidence for fighting at other contemporary centers and a double-palisade was destroyed in a fire around the time of the depopulation of that precinct of Cahokia (52). More broadly, peak residential disparity was followed by the abandonment of a massive area known as the Vacant Quarter, which has been linked to natural disasters that would have impacted food production (i.e., droughts, floods) (53). Cobb and Butler (54) suggest “warfare and social unrest may have hastened the exodus,” and “climatic deterioration… may have exacerbated a competitive and often hostile political environment that rendered the region less hospitable.” We speculate that, when faced with poor conditions for farming and the absence of limits on land, residential disparity reversed its upward trend and more collectively oriented settlement strategies re-emerged.

What we learned here is that to understand the relationship between war and inequality requires at least two different models. This fact helps explain how scholars have been able find support for two diametrically opposed positions. The GINI Project has offered us a glimpse of these models at different points in human history and helped to identify places and times that need more detailed research. We need to look closely at the emergence of less collective forms of government to understand why they seem to create the possibility for war to ratchet-up inequality. Ratcheting-up is strong and sustained where land, not people, is the limiting factor in food production but not where people and their labor are the limiting factor. Through investigating these trends in the evidence of war and inequality in the past, we hope to encourage an evidence-based discussion of these topics as they relate to the contemporary world and promote peace and equality in the future.

## Materials and Methods

*SI Appendix*, Table S1 summarizes residential units and sites coded as having the presence or absence of fortifications. Gini coefficient values were derived from residential unit sizes [(TotalAreaHouse), m^2^] and recorded in the site level GINI Project Database (see ref. 22). This same data was the source for attributes including the presence/absence of fortification [IsFortified], governance [PolityGov], and agriculture [Land-Labor]. The site-level database includes settlements with at least five penecontemporaneous residential units in the residential unit GINI Project Database. Site-level information on fortifications in Southwest Asia was collected separately (*SI Appendix*, Table S2, see *SI Appendix* for more on classification and data). Average residential unit sizes were derived from the residential unit GINI Project Database (*SI Appendix*, Table S3).

### Limitations and Sources of Error

#### Geographic and temporal coverage.

The GINI Project Database was created with existing and available appropriate datasets. We therefore acknowledge that there will be unknown systematic biases in favor of, or against, data from certain kinds of fortified sites for reasons best understood in terms of the local history of archaeological research. The ideal dataset includes both unfortified and fortified sites occupied within the same period of time. However, in some times and places, we have good coverage for unfortified sites but few examples of fortified sites. In others, the opposite is true, making meaningful comparison impossible. This could be sampling bias but may also reflect periods where fortification was ubiquitous (i.e., all settlements were fortified) or when fortification was simply not practiced. Samples from Africa, Mesoamerica, Oceania, and South America are generally small, represented by 16 or fewer fortified sites with >5 residential-unit measurements (*SI Appendix*, Table S1). Temporal coverage is uneven, and there are no early fortifications in the Western Hemisphere sample. We found this unevenness especially problematic in comparing time before/after plant cultivation became common (see ref. 25), and we therefore report results in terms of absolute date estimates (database variable: [Date]).

#### Unindexed variability.

Given such a broad cross-section of societies, we accept that there are likely additional metrics that, if known, might change how we interpret conflict and/or residential disparity. To pick two simple examples, conflict cannot always be expected to map neatly onto the construction of fortifications, and there will be nonmaterial and material factors—such as debt or prestige goods—that may, or may not, map onto residential unit size. Accepting these limitations, we note that because both residences and fortifications are fixed in place, and the fact that this is a global database, we are more likely to be capturing some, if not all, relevant variation. We also note that houses were not coded in terms of their placement in a settlement relative to the location of fortifications.

## Supplementary Material

Appendix 01 (PDF)

## Data Availability

All scripts for replicating the analyses and reproducing main and supplementary figures are provided as an R script in *SI Appendix*. Data archived in the Digital Archaeological Record (tDAR) (55).
