# Supplementary material for: War both reduced and increased inequality over the past ten thousand years
Source: Proc Natl Acad Sci U S A. 2025 Apr 14;122(16):e2400695121. doi: 10.1073/pnas.2400695121 (PMC12037038; doi:10.1073/pnas.2400695121)
Supplement: Supplementary file 1 — Appendix 01 (PDF) [file pnas.2400695121.sapp.pdf]

PNAS

Supporting Information for

War Both Reduced and Increased Inequality Over the Past Ten Thousand Years

Mark D. McCoy, Jennifer Birch, Shadreck Chirikure, Pablo Cruz, Adam S. Green, Detlef Gronenborn, Dan Lawrence, and Paul Roscoe

Corresponding author:
Mark D. McCoy
Email: mark.mccoy@fsu.edu

This PDF file includes:

Supporting text
Tables S1 to S4
SI References

Rscript for Reproducing Figures

Figure 2

```
ggplot(data = Site)+geom_point(mapping = aes(x = Date, y = Gini, color = IsFortified))+
geom_smooth(mapping = aes(x = Date, y = Gini, color = IsFortified))+scale_color_brewer(palette
= "Set2")+theme_bw()
```

Figure 3a

```
ggplot(data = Site[Site$Region == "W Asia and Cyprus",])+geom_point(mapping = aes(x = Date,
y = Gini, color = IsFortified))+ geom_smooth(mapping = aes(x = Date, y = Gini, color =
IsFortified))+scale_color_brewer(palette = "Set2")+theme_bw()
```

Figure 3b

```
ggplot(data = Site[Site$Region == "Southeast NA",])+geom_point(mapping = aes(x = Date, y =
Gini, color = IsFortified))+ geom_smooth(mapping = aes(x = Date, y = Gini, color =
IsFortified))+scale_color_brewer(palette = "Set2")+theme_bw()
```

Metadata for Table S2.

Bigregion. See GINI Project Database metadata for more detail.

Region. See GINI Project Database metadata for more detail.

Subregion. See GINI Project Database metadata for more detail.

Subarea. See GINI Project Database metadata for more detail.

Site. See GINI Project Database metadata for more detail.

SiteID See GINI Project Database metadata for more detail.

Fortified? Are fortifications present (yes) or absent (no).

Fort_type. Text description of fortification type (e.g., External wall only (double))

Fort_code 1=No fortification, 2= External wall only (single), 3= External wall only (double) , 4=Internal walls only, 5= External and internal walls.

Comment. Description of evidence used to classify site.

Table S1. Summary of Variable [IsFortified] in GINI Project Database. This table includes only sites with size information on more than 5 residential units.

	Residential Units				Sites			
	Unfortified (0)	Fortified (1)	NA	Grand Total	Unfortified (0)	Fortified (1)	NA	Grand Total
Africa	47	297	306	512	1	4	10	15
Asia	3591	2762	2751	8594	101	63	101	265
Europe	8298	3278	5070	12287	173	73	139	385
Mesoamerica	5956	4105	5061	14153	37	8	110	155
North America	4215	1181	950	5297	187	47	44	278
Oceania	986	487	0	1473	16	8		24
South America	2804	1262	59	3870	36	16	2	54
	25897	13372	14197	53466	551	219	406	1176

Table S2. Classification of Defenses at Sites in Southwest Asia

Bigregion	Region	Subregion	Subarea	Site	SiteID	Fortified?	Fort_type	Fort_code	Comment
Asia	W Asia and Cyprus	Levant	Levant	Khirbet al-Lahun	1400	Yes	External wall only (double)	3	A "fortification wall" consisting of two parallel walls at a distance of approximately 2 m from each other surrounded the village
Asia	W Asia and Cyprus	Anatolia	Anatolia	Aşıklı Höyük	1401	No	No fortification	1	Clustered neighborhoods in Aşıklı Höyük and at Çatalhöyük are best explained as defense strategy. However, the perimeter wall extends over a small area only. It has been argued that its trajectory was one of 's-shaped' curvatures (ibid.), but this interpretation is unconvincing. Thus, the evidence for a perimeter wall is problematic.
Asia	W Asia and Cyprus	Levant	Levant	Beer Resisim	1402	No	No fortification	1	N/A
Asia	W Asia and Cyprus	Levant	Levant	Beer-Sheba	1403	Yes	External wall only (double)	3	A two-layer circular fortification composed of an outer (w. 1.30-1.35m) and an inner (w. 1m) wall
Asia	W Asia and Cyprus	Levant	Levant	Beidha	1404	Yes	External wall only (single)	2	Neolithic Village Wall, height ranging from 1.0 to 2.2 m
Asia	W Asia and Cyprus	Mesopotamia	Northern Mesopotamia	Bouqras	1405	No	No fortification	1	N/A
Asia	W Asia and Cyprus	Anatolia	Anatolia	Canhasan III	1406	No	No fortification	1	Clustered neighborhoods are best explained as defense strategy according to some researchers.
Asia	W Asia and Cyprus	Anatolia	Anatolia	Çatalhöyük	1407	No	No fortification	1	Clustered neighborhoods at Çatalhöyük are explained as defense strategy by some researchers.

Asia	W Asia and Cyprus	Anatolia	Anatolia	Çayönü	1408	No	No fortification	1	N/A
Asia	W Asia and Cyprus	Levant	Levant	Gilgal I	1409	No	No fortification	1	N/A
Asia	W Asia and Cyprus	Mesopotamia	Northern Mesopotamia	Habuba Kebira South	1410	Yes	External wall only (double)	3	Two parallel city walls, the inner and thicker one with protruding buttresses at regular distances.
Asia	W Asia and Cyprus	Mesopotamia	Northern Mesopotamia	Haradum	1411	Yes	External wall only (single)	2	3 m thick, perfectly rectilinear city wall, corners occupied by bastions
Asia	W Asia and Cyprus	Anatolia	Anatolia	Kerkenes Dağ	1412	Yes	External and internal walls	5	7 km long circuit of stone walls with seven gates
Asia	W Asia and Cyprus	Cyprus	Cyprus	Khirokitia	1413	Yes	External wall only (single)	2	An earlier irregular wall (Mur 100) following the topography of the site, made of pise and faced with stone on its outer side, and a later wall (Mur 284) expanding the site westwards 2.5 m thick, built of stone and interlaid with mud mortar
Asia	W Asia and Cyprus	Anatolia	Anatolia	Kültepe-Kanesh	1414	Yes	External and internal walls	5	Circular circuit wall surrounding the lower town
Asia	W Asia and Cyprus	Cyprus	Cyprus	Marki Alonia	1415	No	No fortification	1	N/A
Asia	W Asia and Cyprus	Levant	Levant	Nahal Oren	1416	No	No fortification	1	N/A
Asia	W Asia and Cyprus	Mesopotamia	Northern Mesopotamia	Nuzi	1417	Yes	External and internal walls	5	Rectangular Town wall 200m in length and width
Asia	W Asia and Cyprus	Levant	Levant	Sidon-Dakerman	1418	Yes	External wall only (single)	2	A stone fence partly encircled the site
Asia	W Asia and Cyprus	Mesopotamia	Northern Mesopotamia	Tell al-Raqā'i	1419	No	No fortification	1	N/A

Asia	W Asia and Cyprus	Mesopotamia	Southern Mesopotamia	Tell Asmar	1420	Yes	External wall only (single)	2	Early Dynastic Town Wall, roughly 7 meters in thickness and made from mudbrick
Asia	W Asia and Cyprus	Levant	Levant	Tell Beit Mirsim	1421	Yes	External wall only (double)	3	Circular Casemate city wall, averaging 1.55 m in width
Asia	W Asia and Cyprus	Levant	Levant	Tell el-Far'ah (North)	1422	No	No fortification	1	"The excavator, de Vaux, suggested that the fortification system of the MB II city was re-utlized in the Iron Age. This assumption is contradicted by the many house walls of all phases of the Iron Age which were built over the line of the old city wall. Furthermore, nowhere are walls integrated into nor do they abut on the old city wall."(Herzog 1997, 216-7) (1)
Asia	W Asia and Cyprus	Levant	Levant	Tell en-Nasbeh	1423	Yes	External wall only (double)	3	Oval Casemate city wall, 4 m wide, constructed of roughly dressed stones and has irregularly-spaced offsets and insets.
Asia	W Asia and Cyprus	Levant	Levant	Tell es Sa'idiyeh	1424	Yes	External wall only (single)	2	Mudbrick city wall, averaging 1.5 m in width with a series of buttresses or projections, 1m. by 1m., at regular intervals on the outside
Asia	W Asia and Cyprus	Mesopotamia	Souther Mesopotamia	Tell es Sawwan	1425	Yes	External wall only (single)	2	Partly surrounded by a wall and ditch, entrance being made on a bent axis approach.
Asia	W Asia and Cyprus	Mesopotamia	Northern Mesopotamia	Tell Halawa A	1426	Yes	External wall only (double)	3	Irregular in form, following the natural topography of the landscape. It consisted of a solid mud-brick wall and a retaining wall with a narrow open space in between, a ditch

									running in front of the retaining wall and an earthen rampart with a steep artificial glacis that connected the ditch and the retaining wall. The walls were constructed of mud bricks resting upon stone footings. A small number of towers or buttresses were attached to the wall at irregular intervals.
Asia	W Asia and Cyprus	Levant	Levant	Tell Masos	1427	No	No fortification	1	N/A
Asia	W Asia and Cyprus	Mesopotamia	Northern Mesopotamia	Tell Sabi Abyad	1428	Yes	External wall only (single)	2	Fortified settlement, a so-called dunnu
Asia	W Asia and Cyprus	Mesopotamia	Northern Mesopotamia	Tell Selenkahiye	1429	Yes	External wall only (double)	3	The defensive wall was equipped with an additional retaining wall, an adjacent ditch and several towers
Asia	W Asia and Cyprus	Mesopotamia	Northern Mesopotamia	Tell Taya	1430	No	No fortification	1	N/A
Asia	W Asia and Cyprus	Mesopotamia	Northern Mesopotamia	Tepe Gawra	1431	No	No fortification	1	N/A
Asia	W Asia and Cyprus	Levant	Levant	Ugarit	1432	Yes	External and internal walls	5	The city was surrounded by a rampart and possibly ashlar fortification walls crossed by the way of a monumental gate although "It is not clear whether the city was still protected by a fortification wall at the very end of the city's existence in the early 12th century B.C.E." (Yon, 2006, 31) (3)
Asia	W Asia and Cyprus	Mesopotamia	Souther Mesopotamia	Ur	1433	Yes	Internal wall only	4	"The nucleus of the city was of course the old walled town of Ur-Nammu. How far the

									walls had been restored it is impossible to say. On the one hand we have the boast of Warad-Sin that he built "a great wall which like a high mountain cannot be undermined", and it is natural to apply this to the town wall, but on the other hand there is no trace of any such construction capping the mud-brick ramp of the Third Dynasty, whereas we do find in the Larsa as in the Kassite period private houses built along its edge, their blank outer walls forming a continuous line and making a good substitute for a military wall of defense. No earlier king of Isin or Larsa claims to have repaired the defenses, and it is probable that none undertook the task; but the townspeople must have taken some steps for their own safety and the most easy and the most economical way of doing so was to adapt their own dwelling-houses to the ends of fortification. It may be that Warad-Sin replaced this make-shift by a real military work, but throughout the earlier part of the period, if our evidence can be trusted, the linked houses had to suffice for the town's protection."

Asia	W Asia and Cyprus	Levant	Levant	Yiftahel	1434	No	No fortification	1	N/A
Asia	W Asia and Cyprus	Anatolia	Anatolia	Zincirli Höyük	1435	Yes	External and internal walls	5	Circular double fortification wall with three gates
Asia	W Asia and Cyprus	Mesopotamia	Northern Mesopotamia	Tell Brak	1436	No	No fortification	1	N/A
Asia	W Asia and Cyprus	Mesopotamia	Northern Mesopotamia	Jerf el Ahmar	1437	No	No fortification	1	N/A
Asia	W Asia and Cyprus	Levant	Levant	Beer-Sheba	1438	Yes	External wall only (double)	3	A two-layer circular fortification composed of an outer (w. 1.30-1.35m) and an inner (w. 1m) wall
Asia	W Asia and Cyprus	Levant	Levant	Beer-Sheba	1439	Yes	External wall only (single)	2	A solid wall made from sun-dried mudbricks, 3.80-3.90 m in thickness for the most part but widening to a maximum of 5.50 m around the city gate
Asia	W Asia and Cyprus	Levant	Levant	Beer-Sheba	1440	Yes	External wall only (single)	2	A solid wall made from sun-dried mudbricks, 3.80-3.90 m in thickness for the most part but widening to a maximum of 5.50 m around the city gate
Asia	W Asia and Cyprus	Anatolia	Anatolia	Çatalhöyük	1441	No	No fortification	1	Clustered neighborhoods at Çatalhöyük are explained as defense strategy by some researchers.
Asia	W Asia and Cyprus	Anatolia	Anatolia	Çatalhöyük	1442	No	No fortification	1	Clustered neighborhoods at Çatalhöyük are explained as defense strategy by some researchers.
Asia	W Asia and Cyprus	Anatolia	Anatolia	Çayönü	1443	No	No fortification	1	N/A
Asia	W Asia and Cyprus	Anatolia	Anatolia	Çayönü	1444	No	No fortification	1	N/A

Asia	W Asia and Cyprus	Anatolia	Anatolia	Çayönü	1445	No	No fortification	1	N/A
Asia	W Asia and Cyprus	Anatolia	Anatolia	Çayönü	1446	No	No fortification	1	N/A
Asia	W Asia and Cyprus	Anatolia	Anatolia	Kültepe-Kanesh	1447	Yes	External and internal walls	5	Circular circuit wall surrounding the lower town
Asia	W Asia and Cyprus	Cyprus	Cyprus	Marki Alonia	1448	No	No fortification	1	N/A
Asia	W Asia and Cyprus	Cyprus	Cyprus	Marki Alonia	1449	No	No fortification	1	N/A
Asia	W Asia and Cyprus	Cyprus	Cyprus	Marki Alonia	1450	No	No fortification	1	N/A
Asia	W Asia and Cyprus	Cyprus	Cyprus	Marki Alonia	1451	No	No fortification	1	N/A
Asia	W Asia and Cyprus	Mesopotamia	Northern Mesopotamia	Tell Asmar	1452	Yes	External wall only (single)	2	Early Dynastic Town Wall, roughly 7 meters in thickness and made from mudbrick
Asia	W Asia and Cyprus	Mesopotamia	Northern Mesopotamia	Tepe Gawra	1453	No	No fortification	1	N/A
Asia	W Asia and Cyprus	Mesopotamia	Northern Mesopotamia	Tell Sabi Abyad	1454	No	No fortification	1	N/A
Asia	W Asia and Cyprus	Mesopotamia	Southern Mesopotamia	Abu Salabikh	1455	Yes	External wall only (single)	2	A semi-rectangular city wall accessible via 7 gates surrounded the Lower City
Asia	W Asia and Cyprus	Levant	Levant	Arad	1456	Yes	External wall only (single)	2	A single contiguous wall between 2 to 2.5 m in thickness encircles the city in an irregular shape. The city wall possesses about 25 circular towers and access into the city is possible through 2-4 large city gates as well as 2-4 postern gates.

Asia	W Asia and Cyprus	Mesopotamia	Northern Mesopotamia	Assur	1457	Yes	External and internal walls	5	Two fortification walls, respectively around the citadel and around the lowertown
Asia	W Asia and Cyprus	Mesopotamia	Southern Mesopotamia	Babylon	1458	Yes	External wall only (single)	2	A single fortification wall, with towers
Asia	W Asia and Cyprus	Levant	Levant	Çatal Höyük (Amuq Valley)	1459	Yes	External wall only (single)	2	A single irregular mudbrick circuit wall roughly 3m in thickness surrounded the tell.
Asia	W Asia and Cyprus	Cyprus	Cyprus	Chlorakas-Palloures	1460	No	No fortification	1	N/A
Asia	W Asia and Cyprus	Mesopotamia	Northern Mesopotamia	Dura Europos	1461	Yes	External wall only (single)	2	Fortifications built in large limestone ashlar blocks, with 26 towers and multiple gates
Asia	W Asia and Cyprus	Cyprus	Cyprus	Episkopi Bamboula	1462	Yes	External wall only (single)	2	Single stone wall around the settlement, only partially excavated by Gisela Walberg
Asia	W Asia and Cyprus	Levant	Levant	Hayonim Cave	1463	No	No fortification	1	N/A
Asia	W Asia and Cyprus	Levant	Levant	Jebel al-Mutawwaq	1464	Yes	External wall only (single)	2	The village is surrounded by an irregular rudimentary stone fence
Asia	W Asia and Cyprus	Levant	Levant	Khirbat al-Mudayna al-Aliya	1465	Yes	External wall only (double)	3	The site is enclosed by a casemate wall measuring between 4.0 and 4.6 m wide, to which most of the site's buildings are directly attached. A 1.1-1.2 m wide outer wall and a 0.8-1.0 m wide inner wall, with casemates of 2.0-2.4 m in between, constitute the casemate wall.
Asia	W Asia and Cyprus	Cyprus	Cyprus	Kissonerga-Mosphilia	1466	No	No fortification	1	N/A
Asia	W Asia and Cyprus	Cyprus	Cyprus	Kissonerga-Mosphilia	1467	No	No fortification	1	N/A

Asia	W Asia and Cyprus	Cyprus	Cyprus	Lemba-Lakkous	1468	No	No fortification	1	N/A
Asia	W Asia and Cyprus	Anatolia	Anatolia	Mersin-Yumuktepe	1469	Yes	Internal wall only	4	Fortified citadel surrounded by an undefended lower town.
Asia	W Asia and Cyprus	Cyprus	Cyprus	Paralimni-Nissia	1470	Yes	External wall only (single)	2	Simple stone wall around the settlement
Asia	W Asia and Cyprus	Cyprus	Cyprus	Parekklesia-Shillourokambos	1471	No	No fortification	1	N/A
Asia	W Asia and Cyprus	Levant	Levant	Qarassa3	1472	No	No fortification	1	N/A
Asia	W Asia and Cyprus	Levant	Levant	Shkārat Msaied	1473	No	No fortification	1	N/A
Asia	W Asia and Cyprus	Cyprus	Cyprus	Sotira-Teppes	1474	Yes	External wall only (single)	2	Part of a stone wall has been excavated along the north edge of the plateau. Although Dikaios was not sure about its interpretation, it closely resembles those of many other Neolithic sites in Cyprus (see. Paralimni Nissia)
Asia	W Asia and Cyprus	Cyprus	Cyprus	Souskiou-Laona	1475	No	No fortification	1	N/A
Asia	W Asia and Cyprus	Mesopotamia	Southern Mesopotamia	Tell Abada	1476	No	No fortification	1	N/A
Asia	W Asia and Cyprus	Mesopotamia	Southern Mesopotamia	Tell Abada	1477	No	No fortification	1	N/A
Asia	W Asia and Cyprus	Mesopotamia	Northern Mesopotamia	Tell Bderi	1478	Yes	External wall only (single)	2	Fortified by a mighty town wall with a glacis in front of it. This walls runs along the foot of the present tell and it was excavated for 17 m.
Asia	W Asia and Cyprus	Mesopotamia	Northern Mesopotamia	Tell Bderi	1479	Yes	External wall only (single)	2	Fortified by a mighty town wall with a glacis in front of it. This walls runs along

									the foot of the present tell and it was excavated for 17 m.
Asia	W Asia and Cyprus	Mesopotamia	Northern Mesopotamia	Tell Bderi	1480	Yes	External wall only (single)	2	Fortified by a mighty town wall with a glacis in front of it. This walls runs along the foot of the present tell and it was excavated for 17 m.
Asia	W Asia and Cyprus	Mesopotamia	Northern Mesopotamia	Tell Bderi	1481	Yes	External wall only (single)	2	Fortified by a mighty town wall with a glacis in front of it. This walls runs along the foot of the present tell and it was excavated for 17 m.
Asia	W Asia and Cyprus	Mesopotamia	Southern Mesopotamia	Tell Harmal	1482	Yes	External wall only (single)	2	A fortification wall, reinforced with towers, measuring 147.3 and 146.9 m in the northwest-to-south direction, and 133 and 98 m in the northeast-to-southwest direction.
Asia	W Asia and Cyprus	Levant	Levant	Tell Mastuma	1483	Yes	External wall only (single)	2	An annular building complex which served both as houses and a fortification were constructed in the peripheral area along the edge of the hill top.
Asia	W Asia and Cyprus	Levant	Levant	Tell Mastuma	1484	Yes	External wall only (single)	2	An annular building complex which served both as houses and a fortification were constructed in the peripheral area along the edge of the hill top.
Asia	W Asia and Cyprus	Mesopotamia	Northern Mesopotamia	Tell Melebiye	1485	No	No fortification	1	N/A
Asia	W Asia and Cyprus	Mesopotamia	Northern Mesopotamia	Tell Munbaqa	1486	Yes	External and internal walls	5	The Outer and Inner Towns were each fortified by a rounded irregular wall.

Asia	W Asia and Cyprus	Levant	Levant	Tell Qasile	1487	No	No fortification	1	A broad mudbrick wall (5.5. m) and a glacis of stamped earth is probably associated with stratum XI; while a casemate wall is associated with strata VIII and II (according to Maisler 1951) (2) but there is no mention of fortification structures associated with stratum X.
Asia	W Asia and Cyprus	Levant	Levant	Tell Zheir	1488	Yes	External wall only (single)	2	An irregular defensive perimeter is maintained around the settlement through the arrangement of houses in a chain-like pattern
Asia	W Asia and Cyprus	Mesopotamia	Southern Mesopotamia	Ur	1489	Yes	External and internal walls	5	In addition to an oval mudbrick rampart and burnt-brick battlement combination encircling the site a rectilinear fortification made up of two parallel walls each 3.25 m in thickness was built to separate the Temenos area from the rest of the city.
Asia	W Asia and Cyprus	Levant	Levant	Wadi Faynan 16	1490	No	No fortification	1	N/A
Asia	W Asia and Cyprus	Mesopotamia	Northern Mesopotamia	Tell Sabi Abyad	1491	No	No fortification	1	N/A
Asia	W Asia and Cyprus	Mesopotamia	Northern Mesopotamia	Tell Chuera	1492	Yes	External and internal walls	5	A wall built during period IA/B around the citadel, and a new 8 m thick wall around the lower town (built in phase IC)
Asia	W Asia and Cyprus	Mesopotamia	Northern Mesopotamia	Tell Chuera	1493	Yes	External and internal walls	5	A wall built during period IA/B around the citadel, and a new 8 m thick wall around the lower town (built in phase IC)

Asia	W Asia and Cyprus	Mesopotamia	Northern Mesopotamia	Tell Chuera	1494	Yes	External and internal walls	5	A wall built during period IA/B around the citadel, and a new 8 m thick wall around the lower town (built in phase IC)
Asia	W Asia and Cyprus	Mesopotamia	Northern Mesopotamia	Tell Billa	1495	Yes	External wall only (single)	2	a "Cyclopean" retaining wall, identifiable in some areas (trench at the base of the eastern slope)to reach a height of 4.5 m and as wide as 2.5 m.
Asia	W Asia and Cyprus	Mesopotamia	Northern Mesopotamia	Tell Bazi	1496	Yes	External wall only (single)	2	A series of walls along the slope of the naturally fortified mound. According to the excavators, some of them worked as retaining or terrace walls, while others "clearly served as fortification walls"
Asia	W Asia and Cyprus	Levant	Levant	Abu Salem G 08, Ramat Harif, G VIII	1498	No	No fortification	1	N/A
Asia	W Asia and Cyprus	Anatolia	Anatolia	Aşağı Pınar	1499	Yes	External wall only (single)	2	Stone wall (Steinmauer)
Asia	W Asia and Cyprus	Anatolia	Anatolia	Aşağı Pınar	1500	Yes	External wall only (single)	2	Stone wall (Steinmauer)
Asia	W Asia and Cyprus	Anatolia	Anatolia	Aşağı Pınar	1501	Yes	External wall only (double)	3	Ditch and palisade (Graben, Palisade)
Asia	W Asia and Cyprus	Anatolia	Anatolia	Aşıklı Höyük	1502	Yes	External wall only (single)	2	Wall (Mauer)
Asia	W Asia and Cyprus	Anatolia	Anatolia	Aşıklı Höyük	1503	Yes	External wall only (single)	2	Wall (Mauer)
Asia	W Asia and Cyprus	Anatolia	Anatolia	Aşıklı Höyük	1504	Yes	External wall only (single)	2	Wall (Mauer)
Asia	W Asia and Cyprus	Anatolia	Anatolia	Aşıklı Höyük	1505	Yes	External wall only (single)	2	Wall (Mauer)

Asia	W Asia and Cyprus	Anatolia	Anatolia	Aşıklı Höyük	1506	Yes	External wall only (single)	2	Wall (Mauer)
Asia	W Asia and Cyprus	Anatolia	Anatolia	Aşıklı Höyük	1507	Yes	External wall only (single)	2	Wall (Mauer)
Asia	W Asia and Cyprus	Anatolia	Anatolia	Aşıklı Höyük	1508	Yes	External wall only (single)	2	Wall (Mauer)
Asia	W Asia and Cyprus	Anatolia	Anatolia	Aşıklı Höyük	1509	Yes	External wall only (single)	2	Wall (Mauer)
Asia	W Asia and Cyprus	Anatolia	Anatolia	Aşıklı Höyük	1510	Yes	External wall only (single)	2	Wall (Mauer)
Asia	W Asia and Cyprus	Anatolia	Anatolia	Aşıklı Höyük	1511	Yes	External wall only (single)	2	Wall (Mauer)
Asia	W Asia and Cyprus	Anatolia	Anatolia	Aşıklı Höyük	1512	Yes	External wall only (single)	2	Wall (Mauer)
Asia	W Asia and Cyprus	Anatolia	Anatolia	Büyük Güllücek	1513	No	No fortification	1	N/A
Asia	W Asia and Cyprus	Anatolia	Anatolia	Can Hasan III	1514	No	No fortification	1	N/A
Asia	W Asia and Cyprus	Anatolia	Anatolia	Can Hasan III	1515	No	No fortification	1	N/A
Asia	W Asia and Cyprus	Anatolia	Anatolia	Can Hasan III	1516	No	No fortification	1	N/A
Asia	W Asia and Cyprus	Anatolia	Anatolia	Can Hasan III	1517	No	No fortification	1	N/A
Asia	W Asia and Cyprus	Anatolia	Anatolia	Can Hasan III	1518	No	No fortification	1	N/A
Asia	W Asia and Cyprus	Anatolia	Anatolia	Can Hasan III	1519	No	No fortification	1	N/A
Asia	W Asia and Cyprus	Anatolia	Anatolia	Can Hasan III	1520	No	No fortification	1	N/A

Asia	W Asia and Cyprus	Anatolia	Anatolia	Çavi Tarlası	1521	No	No fortification	1	N/A
Asia	W Asia and Cyprus	Anatolia	Anatolia	Değirmentepe	1522	No	No fortification	1	N/A
Asia	W Asia and Cyprus	Anatolia	Anatolia	Hallan Çemi Tepesi	1523	No	No fortification	1	N/A
Asia	W Asia and Cyprus	Anatolia	Anatolia	Hallan Çemi Tepesi	1524	No	No fortification	1	N/A
Asia	W Asia and Cyprus	Anatolia	Anatolia	Hallan Çemi Tepesi	1525	No	No fortification	1	N/A
Asia	W Asia and Cyprus	Anatolia	Anatolia	Hassek Höyük	1526	Yes	External wall only (single)	2	a 40 m long and 2 m wide circuit wall (Umfassungsmauer)
Asia	W Asia and Cyprus	Anatolia	Anatolia	Hassek Höyük	1527	Yes	External wall only (single)	2	a 40 m long and 2 m wide circuit wall (Umfassungsmauer)
Asia	W Asia and Cyprus	Anatolia	Anatolia	Kuruçay Höyük	1528	No	No fortification	1	N/A
Asia	W Asia and Cyprus	Anatolia	Anatolia	Norşuntepe	1529	No	No fortification	1	N/A
Asia	W Asia and Cyprus	Anatolia	Anatolia	Norşuntepe	1530	No	No fortification	1	N/A
Asia	W Asia and Cyprus	Anatolia	Anatolia	Norşuntepe	1531	No	No fortification	1	N/A
Asia	W Asia and Cyprus	Anatolia	Anatolia	Norşuntepe	1532	No	No fortification	1	N/A
Asia	W Asia and Cyprus	Anatolia	Anatolia	Norşuntepe	1533	No	No fortification	1	N/A
Asia	W Asia and Cyprus	Anatolia	Anatolia	Norşuntepe	1534	No	No fortification	1	N/A
Asia	W Asia and Cyprus	Anatolia	Anatolia	Norşuntepe	1535	No	No fortification	1	N/A

Asia	W Asia and Cyprus	Anatolia	Anatolia	Norşuntepe	1536	No	No fortification	1	N/A
Asia	W Asia and Cyprus	Anatolia	Anatolia	Norşuntepe	1537	No	No fortification	1	N/A
Asia	W Asia and Cyprus	Anatolia	Anatolia	Norşuntepe	1538	No	No fortification	1	N/A
Asia	W Asia and Cyprus	Anatolia	Anatolia	Norşuntepe	1539	No	No fortification	1	N/A
Asia	Central Asia	Turkmenistan		Akča-Tepe	1540	Yes	External wall only (single)	2	4.5 m large wall made of mudbricks (breite Lehmmauer)
Asia	Central Asia	Turkmenistan		Altyn-Tepe	1541	No	No fortification	1	N/A
Asia	Central Asia	Turkmenistan		Altyn-Tepe	1542	No	No fortification	1	N/A
Asia	Central Asia	Turkmenistan		Chagylly	1543	No	No fortification	1	N/A
Asia	Central Asia	Turkmenistan		Čopan-Tepe	1544	No	No fortification	1	N/A
Asia	Central Asia	Turkmenistan		Džejtun	1545	No	No fortification	1	N/A
Asia	Central Asia	Iran		Ganj Dareh	1546	No	No fortification	1	N/A
Asia	Central Asia	Afghanistan		Mundigak	1547	No	No fortification	1	N/A
Asia	Central Asia	Afghanistan		Mundigak	1548	No	No fortification	1	N/A
Asia	Central Asia	Afghanistan		Mundigak	1549	No	No fortification	1	N/A

Table S3. Frequency of Fortification by Residential Unit Size ([TotalAreaHouse])

		Smallest Q1	Q2	Q3	Largest Q4	
Asia	unfortified (n sites)	35	31	25	10	101
	fortified (n sites)	6	9	16	31	62
	unfortified (% sites)	85%	78%	61%	24%	
	fortified (% sites)	15%	23%	39%	76%	
Southwest Asia (W Asia and Cyprus)	unfortified (n sites)	19	11	6	4	40
	fortified (n sites)	2	10	15	17	44
	unfortified (% sites)	90%	52%	29%	19%	
	fortified (% sites)	10%	48%	71%	81%	
North America	unfortified (n sites)	134	134	125	106	499
	fortified (n sites)	6	7	15	35	63
	unfortified (% sites)	96%	95%	89%	75%	
	fortified (% sites)	4%	5%	11%	25%	
Southeast North America	unfortified (n sites)	25	24	20	12	81
	fortified (n sites)	0	1	4	11	16
	unfortified (% sites)	100%	96%	83%	52%	
	fortified (% sites)	0%	4%	17%	48%	

Table S4. Examples of Classification of Settlements as Fortified

	Minimum Threshold for Classification as Fortified
Africa	Southern Africa – presence of drystone walls enclosing houses or settlements. East Africa – presence of walls making up Swahili settlements like Kilwa. West Africa – presence of ramparts, and ditches surrounding settlements.
Asia	West Asia – presence of substantial wall surrounding all or part of the site (substantial meaning greater width and length than standard domestic architecture). These are generally either baked or unfired mud brick.
Europe	Sites, or parts of sites, enclosed by palisades and/or ditch and rampart and/or stone walls. Roman Britain – defensive walls surrounding the core area of the settlement, and often all of it.
Mesoamerica	Maya- Sites were classified as fortified based on the presence of a wall or ditch and rampart surrounding the central precinct (or beyond, as is the case for Tikal)
North America	Settlements enclosed by a palisade on all sides or on those sides not protected by topography, e.g., steep breaks in slope.
Oceania	Site classification by previous researchers as fortified (in New Zealand, pa). For ethnographic examples, settlement reported as fortified (New Guinea).
South America	Occupations identified by local populations and toponymy as “pukara” (in Quechua and Aymara: fortress). Location in elevated reliefs and with natural barriers (rocks and stromatolite blocks), existence of buildings and perimeter walls. Variable quantity of sling stones on the surface below the sites.

SI References

1. Z. Herzog, *Archaeology of the City: Urban planning in ancient Israel and its social implications* (Graphit Press, 1997).
2. B. Maisler, The Excavation of Tell Qasile. *The Biblical Archaeologist* **14**(2), (1951), pp.43-49.
3. M. Yon, *The City of Ugarit at Tell Ras Shamra* (Eisenbrauns, 2006).
